# Association of Metabolically Healthy and Unhealthy Obesity Phenotypes with Oxidative Stress Parameters and Telomere Length in Healthy Young Adult Men. Analysis of the MAGNETIC Study

**DOI:** 10.3390/antiox10010093

**Published:** 2021-01-11

**Authors:** Mateusz Lejawa, Kamila Osadnik, Tadeusz Osadnik, Natalia Pawlas

**Affiliations:** 1 Department of Pharmacology, Faculty of Medical Sciences in Zabrze, Jordana 38, 41-808 Zabrze, Medical University of Silesia, 40-055 Katowice, Poland; kosadnik@sum.edu.pl (K.O.); tadeusz.osadnik@sum.edu.pl (T.O.); natalia.pawlas@sum.edu.pl (N.P.); 2 2nd Department of Cardiology and Angiology, Silesian Center for Heart Diseases, Marii Skłodowskiej-Curie 9, 41-800 Zabrze, Poland

**Keywords:** metabolically healthy obesity, metabolically unhealthy obesity, oxidative stress, telomere length

## Abstract

Obesity is a significant factor related to metabolic disturbances that can lead to metabolic syndrome (MetS). Metabolic dysregulation causes oxidative stress, which affects telomere structure. The current study aimed to evaluate the relationships between telomere length, oxidative stress and the metabolically healthy and unhealthy phenotypes in healthy young men. Ninety-eight participants were included in the study (49 healthy slim and 49 obese patients). Study participants were divided into three subgroups according to body mass index and metabolic health. Selected oxidative stress markers were measured in serum. Relative telomere length (rTL) was measured using quantitative polymerase chain reaction. The analysis showed associations between laboratory markers, oxidative stress markers and rTL in metabolically healthy and unhealthy participants. Total oxidation status (TOS), total antioxidant capacity (TAC) and rTL were significantly connected with metabolically unhealthy obesity. TAC was associated with metabolically healthy obesity. Telomeres shorten in patients with metabolic dysregulation related to oxidative stress and obesity linked to MetS. Further studies among young metabolically healthy and unhealthy individuals are needed to determine the pathways related to metabolic disturbances that cause oxidative stress that leads to MetS.

## 1. Introduction

Obesity is one of the most common epidemics today and has become one of the greatest health problems of this century [1]. Adverse changes in the lifestyles of modern people in the form of, e.g., increased consumption of high-calorie foods and decreased physical activity, are responsible for the growing number of obese people in society [2]. Suboptimal body weight is a key risk factor in diseases such as type 2 diabetes, dyslipidemia, hypertension and various types of cancer [3,4]. Obesity is usually related to metabolic syndrome (MetS), which is characterized by elevated blood pressure and high triglyceride and glucose levels with concomitant low high-density lipoprotein cholesterol levels, reflecting profound metabolic disturbance [5]. Obesity and MetS are associated with morbidity and mortality mainly due to cardiovascular diseases (CVDs), as well as with abnormal health conditions [6]. Among obese people, there are some that, despite excessive body weight, show no signs of metabolic disorders. Such people are called metabolically healthy obese (MHO). According to numerous studies, MHO individuals are free from obesity-related metabolic disorders, have a lower risk of developing CVDs and show lower mortality than metabolically unhealthy obese (MUO) individuals [7].

Telomere length (TL) is considered a potential marker of cellular aging, and TL shortening is associated with obesity, hypertension and increased risks of morbidity and mortality [8,9,10,11,12]. Telomeres are chromosome terminals composed of conserved oligomeric nucleotide sequences. They are composed of DNA tandem repeats (5′-TTAGGG-3′ in mammals) and associated proteins [13]. The effect of telomeres shortening with age plays an essential role in the aging of cells [14,15]. This implies the potential application of the measurement of telomere length as a biomarker of chronic oxidative stress (OS) [16]. OS is considered a disproportionate relationship between the production of reactive oxygen species (ROS) and their removal by the antioxidant system and is also a factor involved in cell aging [14]. Cell aging can be induced by the intracellular program associated with changes in telomere structure (called uncapping), by their shortening and by environmental factors, the most important of which is oxidative stress [17].

The aim of the present study was to determine the association between telomere length, oxidative stress and metabolically healthy and unhealthy phenotypes in healthy young men.

## 2. Materials and Methods

### 2.1. Study Population

The study group included participants recruited for the MAGNETIC (Metabolic and Genetic Profiling of Young Adults with and without a Family History of Premature Coronary Heart Disease) project. The MAGNETIC project is a case-control study that aims to analyze the classical and genetic risk factors for coronary artery disease (CAD) in healthy young adults with and without a familial history of premature coronary artery disease (P-CAD). For the project’s purpose, we included descendants of patients hospitalized at the Silesian Center for Heart Diseases (2010–2017) and a control group from healthy volunteers. Detailed study protocol has been described in previous articles [18,19].

All study participants, along with a qualified interviewer, filled out a detailed, standardized questionnaire encompassing sociodemographic information, cigarette or alcohol consumption, physical activity, sleep habits and questions regarding family history of CVDs or diabetes mellitus (DM).

From the participants of the MAGNETIC project, we selected 49 healthy slim individuals without MetS and 49 obese patients matched by age and cigarette smoking. The selected population was divided into three subgroups according to body mass index (BMI) (body mass [kg]/body height [m]^2^) and metabolic health: metabolically healthy normal weight (MHNW) (*n* = 49), metabolically healthy obese (MHO) (*n* = 27) and metabolically unhealthy obese (MUO) (*n* = 22). Obesity was defined according to the current World Health Organization (WHO) classification as BMI ≥ 30 kg/m^2^. Metabolic health was defined according to the 2009 International Diabetes Federation (IDF) [20] and the third report of the National Cholesterol Education Program Adult Treatment Panel III (NCEP-ATP III) (Table 1) [21]. Anthropometric measurements (height, weight, waist circumference and hip circumference), systolic blood pressure (SBP) and diastolic blood pressure (DBP) measurements were obtained during the first appointment at the Silesian Centre for Heart Disease.

### 2.2. Laboratory Measurements

Venous blood samples from each participant were collected between 7 AM and 9 AM, approx. 8–10 h after their last meal using S-Monovette tubes coated with EDTA (SarstedtAG&Co. KG., Nümbrecht, Germany) to obtain whole blood or plasma, S-Monovette tubes with a clotting activator (SarstedtAG&Co. KG., Nümbrecht, Germany) to obtain serum and S-Monovette tubes with 3.1% sodium citrate (SarstedtAG&Co. KG., Nümbrecht, Germany) for fibrinogen content. The blood samples were centrifuged at 1500 rpm for 10 min at 4 °C for serum or 3000 rpm for 10 min at 4 °C for plasma. The fibrinogen content was determined using a BCS XP analyzer (Siemens Healthcare, Erlangen, Germany). Total cholesterol (TC), low-density lipoprotein cholesterol (LDL-C), high-density lipoprotein cholesterol (HDL-C), triglycerides (TG), apolipoprotein A1 (apoA1), apolipoprotein B (apoB), lipoprotein(a) (Lp(a)), alkaline phosphatase (ALP), alanine transaminase (ALT), aspartate transaminase (AST), gamma-glutamyltransferase (GGT), lactate dehydrogenase (LDH), high-sensitivity C-reactive protein (hsCRP), glucose, bilirubin, uric acid, concentrations in serum, and glycated hemoglobin (HbA1c) levels in whole blood were measured on a Cobas 6000 analyzer (Roche Diagnostics, Indianapolis, IN, USA) using kits from Roche (Roche Diagnostics, Indianapolis, IN, USA). After biochemistry testing, the remnants of serum, plasma and blood were stored at −80 °C for later assessment.

### 2.3. Measurement of Relative Telomere Length

The relative telomere length (rTL) was measured in peripheral leukocytes in whole blood because blood is a very easily accessible biological material and whole blood rTL positively correlates with tissue-specific rTL measurements [22].

DNA was isolated from peripheral whole blood using a MagCore^®^ HF 16 automated nucleic acid extractor (RBC Bioscience, New Taipei City, Taiwan) and MagCore^®^ Genomic DNA Whole Blood Kit according to the manufacturer’s instructions. The DNA samples were then diluted with sterile nuclease-free water (Invitrogen, Waltham, MA, USA) to a concentration of 10 ng/µL and stored at −20 °C until analysis. Determination of the rTL was performed using quantitative polymerase chain reaction (qPCR), as described by Cawthon [23]. The rTL was determined in relation to a single copy gene (hemoglobin beta chain, HBG). To ensure quality control during reactions, a standard curve of diluted reference DNA (the same DNA sample for all runs), calibrator DNA and no template controls (NTC) were included in each run. Each sample and standard curve of reference DNA (pooled DNA samples, 1.16–74 ng of template) were run in triplicate. The primer sequences (written 5′->3′) obtained from Cawthon’s manuscript (2002) were as follows: telomere forward, GGTTTTTGAGGGTGAGGGTGAGGGTGAGGGTGAGGGT; telomere reverse, TCCC- GACTATCCCTATCCCTATCCCTATCCCTATCCCTA; HBG forward, GCTTC- TGACACAACTGTGTTCACTAGC; and HBG reverse, CACCAACTTCATCCACGTTCACC. The qPCR was performed on a real-time PCR machine (QuantStudio 6, Applied Biosystems, Foster City, CA, USA). For each sample, two reactions were performed (to amplify a single copy gene HGB and for telomeric sequence). Both reactions were performed in a final volume of 10 µL containing 20 ng DNA, 5 µL of Sensitive RT HS-PCR Mix SYBR (A&ABiotechnology, Gdynia, Poland) (containing Taq DNA polymerase, MgCl_2_, dNTP mix and 2× conc. reaction buffer with SYBR Green dye), forward and reverse primers (0.10 μM each for telomeres or 0.20 μM each for HBG) and sterile nuclease-free water. The thermal cycling profile for telomere amplification was 95 °C for 3 min, followed by 30 cycles of 95 °C for 20 s, 58 °C for 1 min and 72 °C for 30 s with a final melting step. The melting curve was performed with 1 cycle of 20 s at 95 °C and 1 min at 72 °C and 95 °C, with a temperature ramp of 0.05 °C/s. The temperature profile for HBG amplification was as follows: 95 °C for 3 min, followed by 35 cycles of 95 °C for 20 s, 58 °C for 30 s and 72 °C for 15 s, with a final melting step. The melting curve was performed with 1 cycle of 20 sec at 95 °C and 60 s at 72 °C, and 95 °C, with a temperature ramp of 0.05 °C/s. The R^2^ for each standard curve was >0.95. Standard deviations for Ct values in triplicate were accepted at <0.2. The inter-assay coefficients of variability for different runs were 4.5% based on 5 runs for telomeres and 1.9% based on 5 runs for HBG. The intra-assay coefficients of variability for different runs were 1.0% based on runs for telomeres and 0.5% based on runs for HBG. Data were analyzed using the same threshold for all samples in QuantStudio real-time PCR software, and then the results were exported to an Excel sheet. The ratio of the telomere (T) product to the HBG product (single copy gene (S)) was calculated using the following formula, which describes the T/S of one sample relative to the T/S of the other as 2^−(ΔCt1-ΔCt2)^ = 2^−ΔΔCt^.

### 2.4. Measurement of Oxidative Stress Markers

Total antioxidant capacity (TAC) and total oxidation status (TOS) were measured in serum according to a protocol developed by Erel [24,25]. The TOS results are expressed as μmol/L, and the TAC results are expressed as mmol/L. The oxidative stress index (OSI) was calculated as the percentage ratio of TOS to TAC. The level of malondialdehyde (MDA) in serum was measured fluorometrically as a thiobarbituric acid reactive substance according to a protocol developed by Ohkawa et al. [26] with modifications. MDA values are expressed as μmol/L.

The level of lipid hydroperoxides (LHPs) in serum was measured according to a protocol developed by Arab and Steghens [27]. The LPH values are expressed as μmol/L. The level of lipofuscin (LPS) in serum was measured according to a protocol developed by Tsuchida [28]. The LPS values are expressed as relative unit (RUI/L). The level of protein sulfhydryl groups (PSHs) in serum was measured according to a protocol developed by Koster, Biemond, and Swaak [29]. The PSH values are expressed as μmol/g protein. The level of ceruloplasmin (CER) was measured spectrophotometrically in serum according to a protocol developed by Richterich [30]. The CER values are expressed as mg/dL. Determination of the activity of superoxide dismutase (SOD) and isoenzyme CuZn-SOD Mn-SOD was measured in serum according to a protocol developed by Oyanagui [31]. The activity values of SOD, CuZn-SOD, and Mn-SOD are expressed in nitric units (NU/mL).

### 2.5. Statistical Analysis

All statistical analyses were performed using free R software [32]. In view of the outlier value of oxidative stress parameters and/or rTL for 3 study participants that were not biologically plausible, their data records were not included in the analyses. Continuous variables are presented as the median and interquartile range (median [Q_1_–Q_3_]). Categorical variables are presented as absolute (*n*) and relative (%) frequencies. Values of continuous and categorical variables concerning clinical characteristics between groups MHNW, MHO, MUO were compared with appropriate tests for trends—Jonckheere–Terpstra or Cochran–Armitage test and the Kruskal–Wallis test by ranks or Chi-squared test. There were some missing data for waist-to-hip ratio (WHR) (1.02%), SBP/DBP (2.04%), PSH (11.22%), CER (11.22%), TAC (11.22%), TOS (11.22%), OSI (11.22%), LPH (11.22%), SOD/CuZnSOD/MnSOD (11.22%), LPS (11.58%), MDA (13.27%), and rTL (4.21%). The correlation between oxidative stress parameters and clinical characteristics was assessed using the Spearman rank correlation test. Additionally, univariable and multivariable regression analyses were performed. We used a general linear model to evaluate the relationship between oxidative stress parameters and metabolic health status. Before performing correlation and multivariable regression analysis, missing values were imputed using the missForest data imputation algorithm [33]. For all tests, a *p*-value of < 0.05 was considered to be statistically significant.

## 3. Results

### 3.1. Demographic, Anthropometric and Biochemical Characteristics of the Study Population

Anthropometrics and biochemical characteristics between the three groups according to metabolic health are shown in Table 2. We did not observe any differences between groups with respect to age, family history of DM, smoking or sleeping habits. However, the groups were different from each other regarding the family history of P-CAD (*p*-value = 0.03) and low physical activity level (*p*-value = 0.04). We observed a significant trend among the obese groups (MHO and MUO) for anthropometric and biochemical measurements. MUO was associated with higher BMI, WHR, visceral adipose index (VAI), SBP, DBP, TC, LDL-C, TG, apoB, glucose, HbA1c, uric acid, GGT, hsCRP, fibrinogen, and liver enzymes (ALT, AST, LDH) and lower HDL, HDL%, apoA1, bilirubin, and CuZnSOD than MHO, who in turn showed a similar trend toward MHNW.

### 3.2. Evaluation of Telomere Length and Oxidative Stress Markers

The MUO group had a significantly shorter rTL (*p*-value = 0.002) than the metabolically healthy (MHNW and MHO) groups (Table 3). Moreover, TAC, TOS, and serum levels of LPH were significantly higher in the MUO group than in the MHNW and MHO groups. Furthermore, the values for PSH, CER, TOS, LPS, and MDA were lower in the MHO group than in the MHNW and MUO groups.

### 3.3. Connection Between Clinical Parameters, Telomere Length and Oxidative Stress

The correlation analysis (Table 4) showed significant positive correlations between selected oxidative stress parameters and BMI (TAC, TOS, LPH), WHR (CER, TAC, LPH, MDA), VAI (TAC, TOS, LPH), SBP (TAC, LPH), DBP (CER), TC (TAC, TOS, LPH), LDL-C (CER, TAC, TOS, LPH), apoA1 (MnSOD), apoB (CER, TAC, TOS, LPH), TG (TAC, TOS, LPH), hsCRP (CER, TAC, TOS, LPH), glucose (PSH, TOS, LPH, MnSOD), HbA1c (TAC, TOS), uric acid (TAC, TOS, CER, LPH), fibrinogen (CER, TAC), and GGT (TAC, TOS, LPH). There were significant negative correlations found between PSH and age, glucose or TAC and apoA, HDL-C, HDL% or TOS and HDL-C, HDL% or OSI and VAI, TG or LPH and HDL-C, HDL%.

rTL was significantly negatively associated with BMI, VAI, TG, uric acid, and GGT. Additionally, rTL was significantly negatively associated only with LPH parameters that indicated increased oxidative stress.

### 3.4. Relationship Between Telomere Length, Oxidative Stress Markers and Metabolic Health Status.

Univariable regression analyses indicated that MUO was associated with decreased rTL (*p*-value = 0.0001) and increased TAC (*p*-value = < 0.0001) and TOS (*p*-value = 0.0005). Additionally, this analysis showed that MHO and MUO were associated with higher levels of LPH (Table 5).

**Figure 1:**
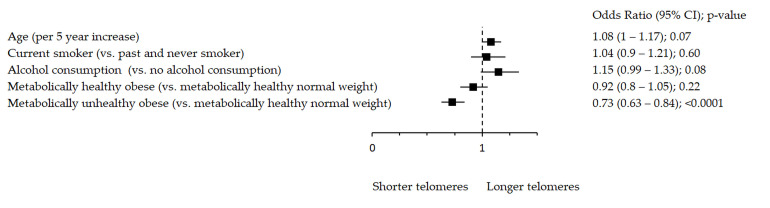
Association between rTL and metabolic health status. The forest plot shows multivariable analyses. The odds ratio with 95% confidence interval (CI) was obtained for precision and adjusted by age, smoking and drinking.

**Figure 2:**
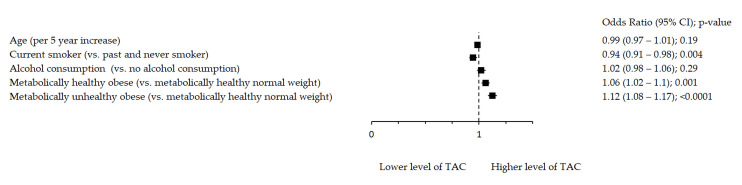
Association between TAC and metabolic health status. The forest plot shows multivariable analyses. The odds ratio with 95% confidence interval (CI) was obtained for precision and adjusted by age, smoking and drinking.

**Figure 3:**
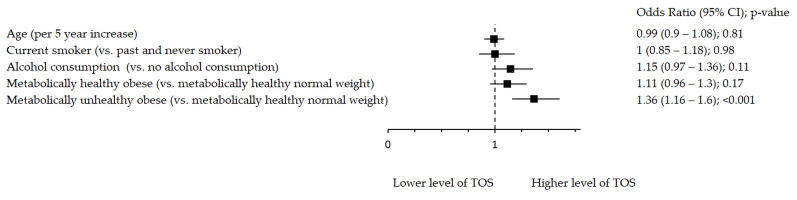
Association between TOS and metabolic health status. The forest plot shows multivariable analyses. The odds ratio with 95% confidence interval (CI) was obtained for precision and adjusted by age, smoking and drinking.

Oxidative stress markers remained significantly associated with metabolic health after adjustment for age, smoking and alcohol consumption (Figure 1, Figure 2 and Figure 3). Figure 1 shows significant inverse association between rTL and MUO as opposed to MHNW, Figure 2 shows that smoking significantly decreases TAC, while both MHO and MUO, independently of the remaining variables, are associated with higher TAC values as compared to MHNW individuals. On the other hand, Figure 3 shows that higher levels of TOS were observed in MUO subjects but not in MHO individuals as compared to MHNW individuals.

## 4. Discussion

The aim of this study was to determine a possible association between oxidative stress, telomere length and metabolic health phenotype. We found that shorter TL was not associated directly with obesity because we did not observe any significant difference in TL between groups that differed in terms of BMI (MHNW vs. MHO). However, it can be seen that the TL is shorter in the presence of MetS abnormalities. The OS, which may cause telomere shortening, plays an important role in the pathophysiology of MetS; therefore, we suggest the use of TL as a possible biomarker of MetS progression [16,34,35,36]. Moreover, TL is considered a good aging marker because it combines both genetic (hereditary) predisposition and the cumulative effect of environmental factors exerted on the organism. Indeed, the combination of metabolic disorders, such as MetS, can be aging related due to display functional decline in tissues, such as the heart and liver, that can increase cardiovascular risk and mortality [37,38]. Many studies have shown that obesity cannot be viewed unambiguously. MHO is a subgroup of patients described as a group with an absence of metabolic disorders such as dyslipidemia, hypertension, insulin resistance, type 2 diabetes and BMIs greater than 30 kg/m^2^. However, it should be added that MHO individuals are noted to have less central and visceral obesity [39,40]. This relationship is also confirmed by the results of our work, and we observed a significant trend towards WHR growth along with a worsened metabolic state. The results of our work are supported by the work of other researchers, where most notably, less ectopic and visceral fat and favorable inflammatory profiles with lower concentrations of inflammatory markers are seen with MHO [41].

It should be emphasized that the prevalence of MHO varies widely depending on how it has been defined. A systematic review by Rey-Lopez et al. evaluated 27 prospective studies and found that prevalence ranged from 6 to 75%, depending on the classification scheme used [2]. It should also be noted that as many as 30% of MHO may convert to the MUO phenotype, resulting in increased cardiovascular risk [41]. This is due to the transition from normal adipose white tissue to that which leads to metabolic derangements. This postulated that once adipocytes reached a threshold capacity for storage, they began to promote insulin resistance with lipotoxicity and release of adipokines [42]. Analyzing the results of our work, we would like to point out that the MHO phenotype shows intermediate features in the assessment of metabolic health conditions, suggesting importance in the interpretation by physicians and an adequate response in lifestyle by patients. In our previous research, we demonstrated that individuals with MetS were more likely to adhere to the Western dietary pattern and have a poor diet quality in comparison to metabolically healthy peers, independent of BMI and WHR. This may imply that diet composition, as an independent factor, plays a pivotal role in increasing metabolic risk. Professional dietary advice should be offered to all metabolically unhealthy patients, regardless of their body mass status [43].

The results of our study indicated that modifiable risk factors for cardiovascular diseases as well as lifestyle significantly affect telomere length. Individuals with shorter TLs exhibit low physical activity levels and unfavorable lipid levels, markers of glucose metabolism, blood pressure, liver markers and higher hsCRP levels. Although the MUO group is slightly older than the others, which undoubtedly affects their TL, it is worth noting that this group shows the greatest attachment to negative risk factors for health. Our study seems to confirm reports of Molli et al., who observed that TL is not directly related to obesity, but that TL becomes shorter with the increase in the number of components of MetS [44]. We did not stratify the individuals in accordance with the variables of MetS as Molli et al. did, but rather based on the definition of metabolic health (IDF/NCEP-ATP III), which qualifies individuals based on the parameters that determine the metabolic health condition.

Environmental factors can explain only a part of the heterogeneity of obesity-induced metabolic alterations between individuals, and the genetic background is an important factor that may explain MHO- and MUO-related obesity [39]. Our results indicate that there is a significant trend towards an increase in the occurrence of a family history of P-CAD among individuals with increasing BMI and MetS, and this trend is inversely related to rTL. Tian et al. found that the mean rate of LTL (leukocyte telomere length) decreased with age and was lower in P-CAD patients than in controls, but the difference was not significant [45]. The results of our work indicated that genetic and environmental factors can explain the heterogeneity of obesity between MHO and MUO, and each factor plays an important role in the aging process. In addition, the results of our work showed that both environmental and genetic factors set the trend of the aging process. The question remains whether modified factors, such as oxidative stress, have an effect on TL. It is well noted that dyslipidemia, hypertension, diabetes, and smoking are traditional risk factors for oxidative stress, and they are together highly correlated with P-CAD, suggesting that oxidative stress and TL shortening may be associated with the disease.

According to some research, obesity increases reactive oxygen species production and causes oxidative stress. Obesity causes disturbances in the Krebs cycle and decoupling of the mitochondrial respiratory chain, which also causes higher ROS production [46]. The results of our work suggest that the accumulation of adipocytes in visceral fat and an increase in other anthropometric variables, such as BMI and WHR, are closely linked with the following oxidative stress markers: TAC, TOS, and LPH, which are inversely correlated with SOD.

Interestingly, the oxidative stress level rises among individuals with obesity and unhealthy metabolic phenotypes. Nevertheless, there is still a lack of data in this field. It is unclear whether the accumulation of abnormalities related to MetS increases the degree of underlying OS, or whether OS is an early manifestation in the pathology of chronic diseases comprising MetS rather than a consequence. Previous studies demonstrated that in obese individuals, insulin resistance may cause inflammation, which is one of the main causes of MetS and type 2 diabetes [47,48].

Oxidative stress plays a key mediatory role in the development and progression of multiple pathophysiological conditions, such as endothelial dysfunction, hypertension and atherosclerotic cardiovascular disease [49]. There is evidence to indicate that a state of chronic low-level inflammation and oxidative stress demonstrates a close link to insulin resistance and MetS [50,51,52]. Some authors suggest that adipose tissue-related oxidative stress causes the progression of MetS by interfering with insulin signal pathway transduction, which can be a major reason for obesity to be a proinflammatory state [35,51]. Following these assumptions, our results support this theory because obesity, not MetS, was the factor that increased the markers of glucose metabolism (glucose and HbA1c). Moreover, Brownlee et al. proved that one of the main reasons for ROS production is the oxidation of excess glucose and fatty acids in adipose tissue [53]. As shown in this study, MHO and MUO subjects had increased LPH levels compared to MHNW individuals. LPHs are the main primary products of lipids, mainly unsaturated lipids, such as omega-3 and omega-6 fatty acids. Increased levels of LPH have been observed in several human inflammatory diseases, including diabetes, hypertension, dyslipidemia and MetS [54,55].

Younus et al. previously mentioned that SOD is an antioxidant enzyme because it is involved in the direct elimination of ROS [56]. It has been suggested that in response to oxidative stress, cells increase the production of this enzyme to protect mitochondria from oxidative damage. The enzyme acts as a good therapeutic agent against reactive oxygen species-mediated inflammatory diseases, such as diabetes [57,58,59,60]. We found inverse correlations between obesity and SOD levels in the blood. This relationship is even stronger in obese individuals with metabolically unhealthy phenotypes. Yubero-Serrano et al. mentioned that SOD activity is individual and is affected only by intrinsic factors, such as genetic factors [61].

Previous studies have also revealed that GGT activity is a major determinant of redox state, and elevation of this enzyme was significantly associated with MetS [62,63]. On the other hand, bilirubin is an antioxidant [64]. Our study of young MHNW vs. young MHO and MUO revealed that significant elevation in GGT activity and a reduced level of bilirubin is associated with the generation of ROS with worsening of metabolic health. Giral et al. obtained similar results [65]. The link between elevated GGT levels and MS was proposed by Stranges et al. and by Andre et al., who also suggested that GGT is associated with hypertriglyceridemia and obesity and represents a marker of insulin resistance [66,67]. Our study also supports the theory that inflammation and oxidative stress may play a role in the development of CVDs, which is frequent in obese individuals with MetS.

Increased serum uric acid concentration is another marker of intracellular and mitochondrial oxidative stress [68]. Yu et al. previously explained that uric acid acts as an antioxidant in the extracellular space; intracellularly, it causes oxidative stress in vascular smooth muscle cells, endothelial cells, adipocytes, renal tubular cells, and hepatocytes, which increases the risk of hepatic fat accumulation and MetS [69]. Our study has shown that the serum uric acid levels increase among the MHNW, MHO, and MUO groups, as well as TOS and the concentration of TC, LDL-C, TG and inversely to HDL and HDL %. Furthermore, Zhang et al. found that not only the amount of fat but also the distribution of fat might be important factors [70]. This trend is in line with our research results. Moreover, Rospleszcz explained that higher serum uric acid levels are strongly associated with increased storage of visceral adipose tissue and hepatic fat [71]. Finally, Han et al. suggested that the path from uric acid level to BMI was more significant than the inverse path [72]. This resulted in the postulation that oxidative stress may be the cause, not the effect, of MetS, which confirms the mentioned thesis, and the uric acid level is a considerable discriminator between the two obesity phenotypes (MHO and MUO).

### Study Limitations

The study population was relatively small, and due to intrinsic limitations of case-control studies, we are not able to establish a cause-effect relationship.

## 5. Conclusions

In summary, our study suggests that telomeres shorten according to the presence of unhealthy obesity phenotype and belong to the main features of MetS. We have also shown that the degree of oxidative stress is influenced by obesity and MetS, but further studies among young healthy and metabolically unhealthy individuals are needed to determine the pathways that lead to MetS.

## Figures and Tables

**Table 1:** Definition of metabolic health (International Diabetes Federation (IDF)/AHA/NHLBI, 2009).

Parameter	Cutoffs
Waist Circumference	Men > 102 cm Women > 88 cm
Blood Pressure	SBP ≥ 130 mm Hg or DBP ≥ 85 mm Hg
	or Use of Antihypertensive Medication *
Triglycerides	≥150 mg/dL
	or Use of Lipid-lowering Medication *
High-density Lipoprotein Cholesterol	Men < 40 mg/dL
	Women < 50 mg/dL
Fasting Glucose	≥100 mg/dL or Drug Treatment for Type 2 Diabetes
Metabolically Healthy	0–2 of the Above Cutoffs
Metabolically Unhealthy	≥3 of the Above Cutoffs

*—None of the subjects were on lipid lowering medication, hypertensive therapy or drug treatment for type 2 diabetes.

**Table 2:** Clinical and demographic characteristics of study participants according to metabolic status.

Characteristics	All	Metabolically Healthy Normal Weight (MHNW)	Metabolically Healthy Obese (MHO)	Metabolically Unhealthy Obese (MUO)	*p*-Value *	*p*-Value **
**N (%)**	95 (100)	47 (49.47)	26 (27.37)	22 (23.16)		
**Age [years]**	31.09 [27.09–31.9]	30.96 [27.10–32.45]	29.23 [26.58–32.48]	31.82 [28.12–33.10]	0.36	0.41
**Current Smoking (vs. Past Smoker or Nonsmoker)**	18 (18.95)	8 (17.02)	4 (15.38)	6 (27.27)	0.52	0.31
**Alcohol consumption**	78 (82.11)	40 (85.11)	20 (76.92)	18 (81.82)	0.68	0.63
**Family History of P-CAD (%)**	53 (55.79)	20 (42.55)	17 (65.39)	16 (72.72)	0.03	0.72
**Family History of DM (%)**	15 (15,79)	6 (12.77)	4 (15.38)	5 (22.73)	0.57	0.52
**Low Physical Activity Level**	42 (44,21)	17 (36.17)	10 (38.46)	15 (68.18)	0.04	0.05
**Less than Six Hours of Sleep per Night During Weekdays**	42 (44.21)	22 (46.80)	12 (46.15)	8 (36.36)	0.70	0.52
**Less than Six Hours of Sleep per Night During the Weekends**	7 (7.37)	3 (6.38)	3 (11.54)	1 (4.55)	0.61	0.35
**BMI [kg/m^2^]**	30.00 [23.38–32.96]	23.36 [21.76–24.00]	31.35 [30.55–33.13]	34.02 [33.03–37.02]	<2.2e-16	0.002
**Waist-to-Hip Ratio (WHR) [cm]**	0.91 [0.83–0.97]	0.84 [0.81–0.89]	0.93 [0.88–0.96]	1.00 [0.97–1.02]	1.444e-10	0.002
**Visceral Adipose Index (VAI)**	0.98 [0.68–1.83]	0.70 [0.49–0.83]	1.34 [1.11–1.79]	3.77 [2.53–5.31]	1.142e-13	0.002
**Systolic Blood Pressure (SBP) [mmHg]**	131.00 [124.75–140.00]	128.00 [120.00–134.00]	136.00 [128.00–145.00]	136.00 [132.00–157.00]	0.0007	0.002
**Diastolic Blood Pressure (DBP) [mmHg]**	82.00 [77.00–89.75]	80.00 [73.50–83.50]	85.50 [76.75–89.50]	90.00 [82.00–95.00]	0.001	0.004
**Total Cholesterol (TC) (mmol/L)**	5.21 [4.48–5.86]	4.80 [4.22–5.35]	5.44 [4.87–6.26]	5.77 [5.04–6.87]	0.0002	0.002
**High-Density Lipoprotein Cholesterol (HDL-C) [mmol/Ll]**	1.31 [1.14–1.59]	1.56 [1.40–1.72]	1.19 [1.15–1.37]	1.01 [0.89–1.15]	4.366e-11	0.002
**HDL%**	27.00 [20.05–32.50]	32.00 [29.00–38.00]	24.00 [20.00–27.00]	17.56 [13.25–21.75]	2.84e-12	0.002
**Low-density LipoProtein Cholesterol (LDL-C) [mmol/L]**	3.27 [2.68–4.11]	2.85 [2.48–3.45]	3.77 [3.02–4.27]	3.75 [3.13–4.27]	0.0001	0.002
**Triglycerides (TG) [mmol/L]**	1.11 [0.79–1.63]	0.80 [0.64–1.03]	1.32 [1.18–1.56]	2.76 [1.94–3.76]	1.543e-14	0.002
**Lipoprotein(a) (Lp(a)) [nmol/L]**	13.00 [4.50–39.00]	17.00 [5.50–44.00]	8.00 [4.25–20.00]	9.50 [3.25–102.75]	0.34	0.18
**Apolipoprotein A1 (apoA1) [g/L**	1.52 [1.42–1.67]	1.57 [1.49–1.70]	1.43 [1.40–1.67]	1.46 [1.32–1.61]	0.02	0.008
**Apolipoprotein B (apoB) [g/L]**	1.01 [0.82–1.21]	0.86 [0.78–1.05]	1.15 [0.93–1.26]	1.23 [1.05–1.42]	4.555e-07	0.002
**Glucose [mmol/L]**	5.10 [4.80–5.45]	5.00 [4.70–5.25]	5.20 [4.85–5.60]	5.35 [5.10–5.90]	0.004	0.002
**Glycated Hemoglobin (HbA1c) [%]**	5.00 [4.80–5.20]	4.90 [4.80–5.20]	5.15 [4.93–5.38]	5.10 [5.00–5.20]	0.01	0.008
**Bilirubin [µmol/L]**	11.80 [8.10–15.60]	12.90 [9.65–17.20]	10.75 [7.90–16.73]	9.05 [6.93–14.03]	0.05	0.006
**Uric Acid [µmol/L]**	361.00 [311.00–398.50]	314.00 [295.00–355.50]	389.00 [359.50–411.25]	403.50 [364.75–439.00]	3.451e-09	0.002
**hsCRP [mg/dL]**	0.91 [0.60–1.69]	0.66 [0.44–1.00]	1.30 [0.64–1.68]	1.73 [1.20–2.25]	2.219e-05	0.002
**Fibrinogen [mg/dL]**	263.00 [233.50–310.00]	248.00 [219.00–292.50]	260.00 [236.25–279.25]	303.00 [260.25–334.00]	0.009	0.002
**Alkaline Phosphatase (ALP) [U/L]**	69.00 [59.00–78.00]	67.00 [54.50–78.50]	66.50 [58.25–76.75]	73.00 [69.00–78.00]	0.20	0.14
**Alanine Transaminase (ALT) [U/L]**	29.00 [18.00–44.00]	19.00 [15.00–27.50]	38.50 [25.75–56.50]	41.00 [34.50–59.00]	1.779e-09	0.001
**Aspartate Transaminase (AST) [U/L]**	23.00 [20.00–30.50]	21.00 [18.00–24.00]	29.00 [22.00–33.75]	26.50 [25.00–33.75]	6.779e-05	0.001
**Gamma-Glutamyltransferase (GGT) [U/L]**	27.00 [17.00–41.50]	17.00 [14.00–24.50]	34.50 [24.25–45.50]	43.50 [33.00–82.75]	7.752e-09	0.001
**Lactate Dehydrogenase (LDH) [U/L]]**	177.00 [156.00–199.50]	162.00 [151.00–179.00]	188.50 [171.25–206.00]	200.50 [178.50–211.50]	1.41e-05	0.001

Continuous variables are presented as the median (interquartile range). Categorical variables are presented as the number of patients (percentages); *—*p*-value of the Kruskal–Wallis rank sum test (continuous variable) or chi-squared test (categorical variable); **—*p*-value of the Jonckheere–Terpstra test (continuous variable) or Cochran–Armitage test (categorical variable); P-CAD—premature coronary artery disease; DM—diabetes mellitus

**Table 3:** Levels of oxidative stress markers among subjects according to metabolic status.

Variable	All	Metabolically Healthy Normal Weight (MHNW)	Metabolically Healthy Obese (MHO)	Metabolically Unhealthy Obese (MUO)	*p*-Value *	*p*-Value **
**Protein Sulfhydryl Groups (PSH) [μmol/g protein]**	4.62 [4.29–4.92]	4.63 [4.32–4.99]	4.59 [4.26–4.74]	4.69 [4.40–4.97]	0.43	0.99
**Ceruloplasmin** **(CER) [mg/dL]**	34.72 [30.84–39.71]	34.21 [30.98–39.59]	33.23 [29.73–37.42]	38.51 [33.60–42.37]	0.07	0.2
**Total Antioxidant Capacity (TAC) [mmol/L]**	0.96 [0.92–1.02]	0.92 [0.89–0.96]	0.97 [0.94–1.02]	1.02 [1.0–1.07]	2.717e-06	0.002
**Total Oxidation** **Status (TOS)** **[umol/L])**	4.19 [3.36–5.21]	4.05 [3.36–4.35]	3.85 [3.27–5.17]	5.29 [4.31–6.04]	0.008	0.01
**Oxidative Stress Index (OSI) (%)**	24 [18.63–28.13]	24 [21.00–28.15]	25 [18.58–30.98]	19 [16.50–25.20]	0.22	0.18
**Lipid** **Hydroperoxides (LPH) [umol/L]**	2.07 [1.70–2.83]	1.71 [1.30–2.02]	2.60 [2.23–3.20]	3.00 [2.60–3.90]	1.041e-09	0.002
**Superoxide Dismutase (SOD) [NU/mL]**	19.31 [18.48, 20.60]	19.54 [19.03–20.61]	19.07 [18.24–19.85]	18.84 [18.07–21.03]	0.17	0.08
**MnSOD (Nu/mL)**	10.79 [10.02–11.60]	10.85 [10.07–11.42]	10.78 [10.03–11.78]	10.58 [10.00–12.12]	0.92	0.63
**CuZnSOD [NU/mL]**	8.48 [7.79–9.24]	8.72 [8.17–9.39]	8.30 [7.46–9.23]	8.16 [7.73–8.90]	0.1	0.04
**Lipofuscin (LPS) [RU/L]**	101.31 [75.70–138.59]	97.81 [71.94–138.66]	94.29 [80.52–149.25]	111.23 [80.04–128.93]	0.89	0.63
**Malondialdehyde (MDA) [umol/L]**	1.42 [1.17–1.93]	1.40 [1.18–1.83]	1.36 [1.00–2.06]	1.61 [1.29–1.98]	0.45	0.55
**Relative Telomere Length (rTL) [relative units]**	0.23 [0.20–0.30]	0.25 [0.21–0.33]	0.23 [0.20–0.27]	0.18 [0.16–0.22]	0.001	0.002

Continuous variables are presented as the median (interquartile range). Categorical variables are presented as the number of patients (percentages); *—*p*-value of the Kruskal–Wallis rank sum test (continuous variable) or chi-squared test (categorical variable); **—*p*-value of the Jonckheere–Terpstra test (continuous variable) or Cochran–Armitage test (categorical variable).

**Table 4:** Correlations between oxidative stress parameters and age, anthropometric measurements and other laboratory findings.

Variable	Protein Sulfhydryl Groups (PSH)	Ceruloplas-min (CER)	Total Antioxidant Capacity (TAC)	Total Oxidation Status (TOS)	Oxidative Stress Index (OSI)	Lipid HyDroperoxides (LPH)	SuperOxide Dismutase (SOD)	Mn SOD	CuZn SOD	Lipofuscin (LPS)	Malondialdehyde (MDA)	Relative Telomere Length (rTL)
**Age**	−0.21 *	0.07	−0.03	−0.04	0.05	−0.03	0.12	0.12	−0.01	0.12	0.15	0.15
**BMI**	−0.14	0.12	0.49 ****	0.27 *	−0.13	0.64 ****	−0.19	−0.08	−0.15	−0.02	0.10	−0.34 ***
**Waist-to-Hip Ratio (WHR)**	−0.03	0.24 *	0.46 ****	0.15	−0.02	0.39 ***	−0.17	−0.04	−0.22 *	0.03	0.25 *	−0.18
**Visceral Adipose Index**	−0.03	0.08	0.52 ****	0.43 ****	−0.26 *	0.61 ****	−0.15	−0.10	−0.08	0.02	0.09	−0.23 *
**Systolic Blood Pressure (SBP)**	0.08	0.07	0.29 *	0.07	−0.02	0.32 **	−0.05	0.12	−0.19	0.005	−0.09	−0.18
**Diastolic Blood Pressure (DBP)**	0.03	0.26 *	0.16	−0.03	0.10	0.19	−0.05	0.12	−0.15	0.03	−0.02	−0.07
**Total Cholesterol (TC)**	−0.14	0.20	0.35 ****	0.24 *	−0.12	0.35 ***	−0.07	0.07	−0.14	−0.03	−0.01	−0.02
**Low-density Lipoprotein Cholesterol (LDL-C)**	−0.13	0.21 *	0.30 **	0.21 *	−0.12	0.31 **	−0.04	0.07	−0.06	0.02	−0.004	0.04
**Apolipoprotein A1 (apoA1)**	0.08	0.05	−0.22 *	−0.10	0.04	−0.22 *	0.18	0.22 *	−0.07	−0.04	−0.04	0.20
**Apolipoprotein B (apoB)**	−0.02	0.22 *	0.42 ****	0.24 *	−0.11	0.41 ****	−0.10	0.07	−0.17	−0.00005	0.01	−0.05
**High-density Lipoprotein Cholesterol (HDL-C)**	−0.04	0.02	−0.45 ****	−0.31 **	0.18	−0.51 ****	0.11	0.10	0.03	−0.002	−0.07	0.26
**HDL%**	0.07	−0.13	−0.51 ****	−0.33 **	0.18	−0.55 ****	0.13	0.02	0.12	−0.02	−0.04	0.19
**Triglycerides (TG)**	0.01	0.08	0.57 ****	0.50 ****	−0.32 **	0.65 ****	−0.09	−0.03	−0.08	0.04	0.04	−0.23 *
**Lipoprotein(a) (Lp(a)**	0.06	0.06	−0.08	−0.11	0.11	−0.20	0.08	0.09	−0.02	−0.05	0.04	0.16
**hsCRP**	−0.02	0.34 ***	0.35 ***	0.32 **	−0.19	0.40 ****	−0.12	−0.10	−0.06	−0.03	0.08	−0.13
**Glucose**	0.21 *	0.05	0.14	0.24 *	−0.20	0.24 *	0.07	0.25 *	−0.19	−0.02	−0.02	0.10
**Glycated Hemoglobin (HbA1c)**	−0.04	0.09 *	0.29 *	0.27 *	−0.14	0.21	−0.01	0.12	−0.10	0.06	0.09	0.20
**Uric Acid**	−0.18	0.22 *	0.73 ****	0.28 *	−0.08	0.46 ****	0.01	0.05	−0.03	0.13	0.07	−0.26 *
**Fibrinogen**	−0.004	0.36 ***	0.24 *	0.24	−0.15	0.17	−0.04	0.01	−0.10	−0.15	−0.02	0.01
**Gamma- Glutamyltransferase (GGT)**	−0.16	0.17	0.49 ****	0.31 **	−0.14	0.50 ****	−0.07	−0.03	−0.07	0.09	0.07	−0.24 *
**Bilirubin**	0.05	0.008	−0.002	−0.18	0.17	−0.22 *	0.15	0.10	0.02	−0.03	0.03	0.06
**Alkaline Phosphatase (ALP)**	0.24 *	0.05	0.13	0.24 *	−0.20	0.23 *	0.17	0.11	0.05	−0.01	−0.07	−0.03
**Alanine Transaminase (ALT)**	−0.16	0.21 *	0.44 ****	0.12	0.01	0.42 ****	−0.18	0.00	−0.20	0.15	0.19	−0.24*
**Aspartate Transaminase (AST)**	−0.10	0.26 *	0.42 ****	0.12	0.01	0.30 **	−0.14	0.03	−0.20	0.01	0.08	−0.19
**Lactate Dehydrogenase (LDH)**	0.002	0.22 *	0.38 ***	0.23 *	−0.13	0.50 ****	−0.03	0.18	−0.26 *	0.07	−0.03	−0.20
**Relative Telomere Length (rTL)**	0.19	0.05	−0.18	0.02	−0.06	−0.35 ***	0.16	0.16	−0.02	−0.05	0.02	-

*p*-value: * < 0.05, ** < 0.01, *** < 0.001, **** < 0.0001.

**Table 5:** Univariate analyses of the effects of oxidative stress markers on metabolic health status.

	Variable	OR	95% CI	*p*-Value
**Univariable Analysis**				
	Log rTL-MHO	0.897	0.777–1.036	0.14
	Log rTL-MUO	0.729	0.626–0.849	0.0001
	Log TAC-MHO	1.063	1.019–1.109	0.006
	Log TAC-MUO	1.117	1.071–1.165	<0.0001
	Log TOS-MHO	1.901	0.913–1.301	0.34
	Log TOS-MUO	1.378	1.158–1.640	0.0005
	Log LPH-MHO	1.622	1.356–1.941	<0.0001
	Log LPH-MUO	1.944	1.630–2.319	<0.0001

OR: odds ratio; CI: 95%: confidence interval.

## Data Availability

The data presented in this study are available on request from the corresponding author.
